# Cross-sectional analysis of socioeconomic drivers of PM2.5 pollution in emerging SAARC economies

**DOI:** 10.1038/s41598-024-67199-z

**Published:** 2024-07-16

**Authors:** Mohammad Musa, Preethu Rahman, Swapan Kumar Saha, Zhe Chen, Muhammad Abu Sufyan Ali, Yanhua Gao

**Affiliations:** 1 School of Business Administration, Xi’an Eurasia University, No. 8 Dongyi Road, Yanta District, Xi’an, 710065 Shaanxi China; 2 International Business School, Shaanxi Normal University, No. 620, West Chang’an Avenue, Chang’an District, Xi’an, 710119 Shaanxi China; 3 Department of Marketing, Xi’an Jiaotong University, Xi’an, 710049 Shaanxi China; 4 College of Business Administration, International University of Business Agriculture and Technology (IUBAT), Dhaka, 1230 Bangladesh; 5 College of Economics, Shenzhen University, Shenzhen, 518055 China; 6 Graduate School of Management, Post Graduate Centre, Management and Science University, University Drive, Off Persiaran Olahraga, Section 13, Shah Alam, 40100 Selangor Malaysia

**Keywords:** SAARC, PM2.5, Socioeconomic-natural-anthropogenic factors, Haze pollution, Environmental economics, Environmental social sciences

## Abstract

Within the intricate interplay of socio-economic, natural and anthropogenic factors, haze pollution stands as a stark emblem of environmental degradation, particularly in the South Asian Association for Regional Cooperation (SAARC) region. Despite significant efforts to mitigate greenhouse gas emissions, several SAARC nations consistently rank among the world’s most polluted. Addressing this critical research gap, this study employs robust econometric methodologies to elucidate the dynamics of haze pollution across SAARC countries from 1998 to 2020. These methodologies include the Pooled Mean Group (PMG) and Augmented Mean Group (AMG) estimator, Panel two-stage least squares (TSLS), Feasible Generalized Least Squares (FGLS) and Dumitrescu–Hurlin (D–H) causality test. The analysis reveals a statistically significant cointegrating relationship between PM2.5 and economic indicators, with economic development and consumption expenditure exhibiting positive associations and rainfall demonstrating a mitigating effect. Furthermore, a bidirectional causality is established between temperature and economic growth, both influencing PM2.5 concentrations. These findings emphasize the crucial role of evidence-based policy strategies in curbing air pollution. Based on these insights, recommendations focus on prioritizing green economic paradigms, intensifying forest conservation efforts, fostering the adoption of eco-friendly energy technologies in manufacturing and proactively implementing climate-sensitive policies. By embracing these recommendations, SAARC nations can formulate comprehensive and sustainable approaches to combat air pollution, paving the way for a healthier atmospheric environment for their citizens.

## Introduction

The SAARC region has experienced substantial environmental degradation, specifically with regard to air quality, due to the interplay of accelerated economic expansion, urbanization and human endeavors. In recent times, these factors have played a significant role in the increase of air pollution, with haze pollution becoming a significant source of concern. There has been a notable surge in public consciousness concerning the matter of haze pollution, the primary pollutant attributed to haze pollution^1,2^. The prevalence of haze pollution is not just an environmental concern; it presents a substantial risk to public health, economic prosperity, and regional stability. Haze pollution is mainly caused by the presence of PM2.5, which refers to particulate matter that has a diameter of less than 2.5 mm. This fine particulate matter has the ability to linger in the atmosphere for long periods of time, exacerbating the detrimental impacts of haze pollution. It is crucial to emphasize the significance of tackling this matter. Exposure to PM2.5 has significant implications for human health, affecting respiratory systems and potentially resulting in long-term illnesses. The decline in public perception of air quality has had a significant impact on the overall quality of life and has the potential to hinder economic activities such as tourism. In addition, the ecological consequences of haze pollution go beyond regional boundaries, impacting ecosystems and biodiversity on a global scale. Addressing haze pollution in the SAARC region is crucial for protecting the health of its citizens, promoting sustainable economic growth, and fostering regional environmental cooperation.

SAARC comprises more than 25% of the global population. Furthermore, this area significantly contributes to the global economy, accounting for around 4% of the total global gross domestic product. Moreover, it constitutes 2% of global product exports and consumes over 25% of the world’s energy. The SAARC region is also accountable for the urbanization of approximately 15% of the world’s population^2,3^. It is evident from the differential growth rates of these factors that the regional GDP is highly energy intensive, which is concerning in terms of the potential for emissions in the region^4^. Considering the intense economic development of the region, SAARC Energy Centre^5^ predicted that the annual GDP of the region, industries, total consumption of goods and associated energy demand would increase by eight percent and seven percent by 2030 because of the area’s intense economic development^2^. The region is currently experiencing significant economic development and is approaching a rapid industrialization process. This has led to an increase in economic growth, consumer spending and energy consumption to meet the demands of emerging industries, urban populations and investments. Consequently, there has been a decline in green and forested areas within the region^2^. According to Brauer^6^, a significant proportion of member nations (62%) are included in the top twenty most polluted countries globally due to haze pollution in this region. Hence, it is crucial to mitigate haze pollution in this region since several variables contribute to the degradation of air quality. Comprehending these elements makes it possible to formulate evidence-based strategies for addressing this issue.

Yang et al.^7^ reported the source of PM pollutants are all kinds of vehicles, dirt and dust from civil construction, broken-off roads, exposed lands, wind corrosion and burning of biomass in the brick fields and city incinerators during the dry season. PM2.5 pollutants are also produced by a variety of sources and components that scholars have extensively studied. Events in the social and economic sphere can influence these sources. A broad categorization may be established between the determinants influencing the accumulation of PM2.5 and those influencing its dispersion. The aggregation, transmission and dispersion of PM2.5 are influenced by several environmental elements, including terrain, wind speed, temperature and precipitation. Conversely, societal concerns are linked to the development of haze pollution^1,8^. Although PM2.5 is emitted by numerous sources, urbanization and land use are unquestionably among the most significant anthropogenic polluters^2,9^. Deserts contribute significantly to PM2.5 concentrations due to their substantial production of sand and dust^10^. In contrast, forests have the capacity to absorb and filter PM2.5 contaminants^11^.

Air pollution, specifically PM2.5, is a complex problem that has multiple causes. This study explores the relationship between PM2.5 and various factors, including GDP, consumer expenditure, forest area, annual temperature and precipitation. These factors are considered as potential drivers of PM2.5 levels. It is of utmost importance to analyze these connections as numerous studies^2,12–14^ have established a correlation between economic growth, as measured by GDP and heightened energy consumption and industrial activity. These activities can be major contributors to PM2.5 emissions, especially in regions undergoing rapid economic growth. Consumer expenditure patterns also have an impact. Studies indicate that higher expenditures on goods that have a significant amount of energy embedded in them, such as manufactured products, have the potential to contribute to the pollution of fine particulate matter (PM2.5) at various stages of their production and transportation^15–17^. Forest cover plays a crucial role in purifying the air by acting as a natural filter for air pollutants. Numerous studies^18–20^ have consistently shown a strong connection between deforestation and increased levels of PM2.5. Ultimately, climate variables such as annual temperature and precipitation have the potential to impact atmospheric conditions that can in turn affect the dispersion of PM2.5^2,21,1^. Gaining a deep understanding of these intricate interactions is essential in order to pinpoint the key factors that contribute to PM2.5 pollution. This knowledge will help guide the development of specific policy interventions aimed at achieving cleaner air. In order to effectively analyze strategies designed to mitigate pollution, it is vital to possess a thorough comprehension of the socio-economic variables linked to PM2.5 pollution^22^. Numerous studies have been undertaken to perform qualitative analyses on the economic aspects that contribute to haze pollution and explore viable strategies for its mitigation^23^. Furthermore, previous research has been dedicated to examining the characteristics that contribute to the presence of PM2.5 pollution and evaluating several strategies for its reduction^2,24^. The inclusion of environmental factors in development plans and strategies is of utmost importance for governments and growth economists, as evidenced by extensive academic study undertaken by Musa et al.^2^ and Ahmed et al.^25^.

Our objective is to assess the collective impact of many possible influencing variables on haze pollution in the region. Before addressing the sternness of SAARC’s haze pollution problem, some questions need to be addressed to clarify our study objectives. (1) Do economic growth activities contribute to the air quality locally and if so, how? (2) What anthropogenic and social activity resulted in the degradation or improvement of air quality? (3) Does the natural environment impact the air quality in this particular region? This study aims to answer these questions and identify the factors that degrade air quality and interact with one another in a significant way in order to improve air quality. Before attaining the objective of decreasing the PM2.5 concentration, it is essential to examine the economic, anthropogenic and environmental factors that affect PM2.5^26^. Prior studies have predominantly concentrated on individual elements such as natural, socioeconomic and anthropogenic factors^2^. Moreover, studies have generally overlooked the impact of both natural and anthropogenic variables on haze. The research has primarily concentrated on economic and social variables, with minimal attention given to environmental aspects^26^. PM2.5 is influenced by natural, social and anthropogenic variables and the utilization of these aspects has been proven to be successful^2^. Numerous facets of our research we believe has the potential to make substantial and significant contributions to the existing body of literature. First, due to the absence of comprehensive and enduring PM2.5 pollution data, particularly in developing nations where monitoring networks are mostly absent, attaining a thorough comprehension of the intricate interplay among many components remains unattainable. The feasibility of utilizing remote sensing techniques for the extraction and calculation of long-term PM2.5 data has been determined as a viable approach for resolving the aforementioned issue. Consequently, a panel data set spanning an extended period was constructed using the PM2.5 geographical information obtained from satellite observations. Second, to the best of our knowledge, the majority of study has primarily examined the impact of individual components, such as those of a natural, anthropogenic, or economic nature, rather than considering their collective and synergistic effects. Very few studies have been undertaken to investigate the effects of various variables on air pollution, namely PM2.5 pollution, within the SAARC area. Third, limited studies have been conducted about the correlation between consumer spending and forest acreage with air pollution in the SAARC region, despite the significant social and human implications associated with these issues. The final phase of this study involves the assessment of economic and human activities in relation to the phenomenon of haze pollution. Thus, this study utilized panel data spanning from 1998 to 2020 to investigate the influence of factors like economic growth, consumer expenditure, forest area, temperature and total rainfall on PM2.5 pollution within the SAARC region.

## Theoretical basis and literature review

The issue of severe haze pollution has garnered significant public interest, with PM2.5 being identified as the primary pollutant responsible for this environmental concern. Numerous researchers have extensively examined the origins and factors that contribute to the production of PM2.5 pollutants. Several studies have identified economic development, energy consumption, construction and urbanization as significant contributors to air pollution^13,27^. Additionally, the use of energy, energy mix and technology also play a role in its impact^9^. Contrary to the assertions made by environmentalists, it is widely acknowledged that energy serves as the major contributor to the emissions of both industrialized and non-industrialized sectors^25^. A causal analysis of economic growth and environmental deterioration is conducted by Samour et al.^28^ and Muhammad et al.^29^ since economic growth and associated social activity are highly dependent on efficient energy use^30^. The current investigation has revealed a favorable association between economic development and the accompanying social activities, as well as the consequent increase in energy consumption and emissions of greenhouse gases. While human activities contribute to the increase in PM2.5 levels, it is evident that the severity of this issue varies in densely populated areas due to natural factors^31^. Several studies have been conducted to investigate the causal link between economic development, energy consumption and environmental degradation in the member countries of the South Asian Association for Regional Cooperation (SAARC). Musa et al.^2^ and Akhmat et al.^32^ have presented empirical findings that substantiate the proposition that energy use significantly contributes to the exacerbation of pollutant emissions. Many scholars argue that there is a negative relationship between economic growth and environmental pollution, which aligns with the theory of the reversed U-shaped EKC theory.

The per capita consumption expenditures serve as an indicator of the consumption patterns and behaviors of individuals residing in a certain area. Cities with higher consumption levels have elevated concentrations of PM2.5 due to the presence of a greater number of homes, autos, urbanization and household appliances^8^. Consequently, a substantial amount of pollution is present in the Earth’s atmosphere. A positive association exists between air pollution and domestic consumption, as shown in both advanced countries and emerging nations, where the latter is seeing a rise in domestic consumption^13^. When examining the environmental effect of household appliances used in everyday life, per capita consumption expenditures provide a more comprehensive perspective compared to GDP per capita. In the context of environmental impact assessment, GDP per capita serves as a metric to gauge the influence of household appliances on the environment. In their study, Ozyilmaz et al.^33^ discuss the impact of public expenditures and their sub-components on environmental pollution in G-7 countries. The study suggests that public spending has a positive impact on reducing environmental pollution. When examining the outcomes of different aspects of public spending, it becomes evident that certain areas such as housing and community amenities, social protection, health expenditure, economic affairs and recreation, culture & religion expenditures contribute to environmental pollution in a detrimental way.

Various control variables typically have a significant impact on environmental pollution. A study by Ji et al.^34^ has provided evidence to support the notion that the absorption of particulate matter by plants serves as a very effective mechanism for air purification, resulting in a substantial reduction in atmospheric particulate matter levels. The study conducted by Begum et al.^35^ utilized time series data to illustrate a clear inverse relationship between forest area and CO2 emissions. In their recent study, Mandal et al.^36^ examined the relationship between soil organic carbon pool, litter carbon and forest carbon storage. The findings of their research indicate a concerning decrease in India’s forest area and its impact on pollution levels. The increase in forest coverage leads to a proportional improvement in the ability to adsorb particulate particles. The study conducted by Ramon et al.^18^ provides evidence that trees act as a natural defense against PM. The study found that implementing low-cost nature-based solutions (NbS) can effectively reduce air pollution and have a positive impact on human health and well-being. It recommends that municipal policies and programs, particularly in areas with high human density and poorly managed green spaces, should include these solutions. Multiple studies have demonstrated the substantial impact of natural variables on haze pollution^37^. These variables encompass several aspects, such as average temperature, rainfall and others^9^. According to Zhang and Cao^38^, the average temperature exerts an impact on the chemical processes involved in the production of pollutants, such as PM2.5, by means of catalytic activity. The phenomenon of clotting, rainfall and aerosols has been seen to facilitate the removal of pollutants from the atmosphere^39,40^.

It is crucial to have a thorough understanding of the socioeconomic factors driving PM2.5 pollution when formulating pollution reduction policies^22^. A group of researchers conducted a qualitative examination of the economic factors behind haze pollution and explored strategies for mitigating it^23^. Meanwhile, another group focused on quantifying the various factors that contribute to haze pollution, including urbanization, technological advancement, energy and environmental legislation, energy consumption and other strategies for mitigating PM2.5^24,27^. In a comprehensive study conducted by Li et al.^41^, the factors driving PM2.5 emissions were thoroughly examined. The findings of the study revealed that economic development, industrialization and urbanization were significant contributors to the long-term increase in PM2.5 pollution. Additionally, Xu et al.^42^ found that various factors such as economic growth, energy intensity, urbanization, coal consumption, personal vehicles and population mass have an impact on PM2.5 pollution. Extensive research on these matters has led growth economists and governments to incorporate environmental factors into the development policy-making process^2^.

In summary, to the best of our knowledge, only a small number of studies have utilized long-term PM2.5 remote sensing data for econometric analysis. Nevertheless, numerous studies have focused on analyzing the sources of PM2.5 pollution. This emphasis is primarily due to the absence of a comprehensive and long-term spatial dataset on PM2.5, as addressed by Wang et al.^43^. A significant amount of research has been conducted on economic, anthropogenic and social variables. However, there has been relatively little focus on the combined impact of natural factors^24^. If research solely focuses on the influencing factors from economic and social perspectives, the empirical results are bound to be inaccurate. Our study aimed to investigate the impact of various factors, both natural and human-induced, on haze pollution that has not been adequately addressed in previous research. Nevertheless, there is already a significant body of research on the impact of these factors on haze pollution. However, it would be beneficial to investigate whether the effects differ when studying them collectively and in specific regions such as SAARC. The research on the impact of economic, anthropogenic and natural factors on haze pollution has always been a primary focus and the scope of this research is continuously expanding. Our aim is to conduct a comprehensive study on the effects of economic growth, consumer expenditure, total rainfall and average temperature on haze pollution in the SAARC region. We will analyze panel data from 1998 to 2020 to determine the extent of their influence.

## Methodology and the model

**Figure 1:**
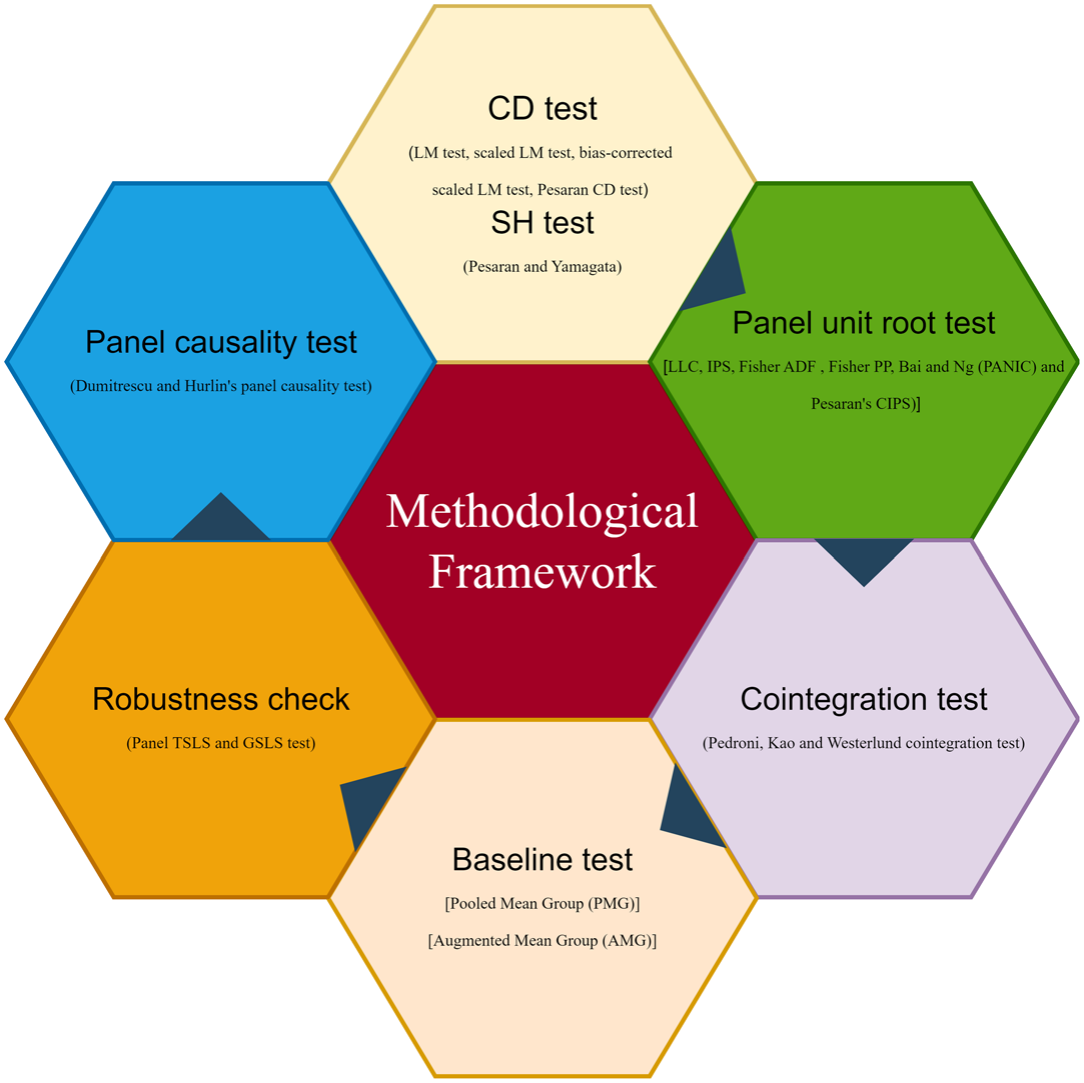
The methodological framework of the study. The figure graphically explains the empirical framework.

This section discusses the study area, data sources and how PM2.5 interacts with other pollutants in the atmosphere and how those interactions affect haze pollution in particular. Furthermore, based on previously identified gaps, these interactions in this study were quantified using a comprehensive and leading set of econometric analysis techniques. This article delineates six pivotal stages in the estimating process. To begin with, In order to assess the presence of heterogeneity, it is essential to perform the CD test as suggested by Pesaran^44^, followed by the slope homogeneity test developed by Pesaran and Yamagata^45^. The next phase entails evaluating the stationarity of each variable. In order to determine the presence of stationarity, a combination of first-generation and second-generation panel unit root tests were employed. The PANIC test, which was established by Bai and Ng^46^ and the CIPS test, suggested by Pesaran^47^, are two widely acknowledged and reliable second-generation unit root tests. In the fourth stage, the cointegration tests for panel data proposed by Pedroni^48^, Kao^49^ and Westerlund^50^ were employed to investigate the presence of cointegrating relationships among the selected variables and assess their correlation. Additionally, we have conducted an empirical analysis using the Pooled Mean Group (PMG) and the panel Augmented Mean Group (AMG) estimators to analyze the dynamic long-run and short-run associations between variables. The research employed the Panel Two-Stage Least Squares (TSLS) and Feasible Generalized Least Squares (FGLS) estimators to assess the reliability of the results and estimate the coefficients and magnitude of the enduring relationships between the independent and dependent variables. The Dumitrescu and Hurlin^51^ non-causality test has been employed to investigate the causative relationship between variables. Figure 1 presents a visual representation of the model requirements.

### Theoretical framework and model specification

This study aims to create a model that considers the influence of socio-economic disparities, natural phenomena and human activity on environmental degradation, specifically focusing on haze pollution. Based on the characteristics of the dependent and independent variables, it seems that the widely used EKC model is the most suitable choice for the given data. In line with the research conducted by Musa et al.^2^ and Sikder et al.^52^, an investigation was carried out on the relations between haze pollution (PM2.5), per capita GDP based on PPP (GDP), consumption expenditure (COEXP), forest area (FA), average annual temperature (TEMP) and total annual rainfall (PREC) in six SAARC countries. The study focused on examining these relationships using a hypothetical EKC model and it is modeled as follows:

1 $$PM2.5_{it} = f\left( {GDP_{it} ,COEXP_{it} ,FA_{it} ,TEMP_{it} ,PREC_{it} } \right),$$

where PM2.5 is the primary explanatory variable. The independent variables are GDP, COEXP, FA, TEMP and PREC. In a linear combination with matching parameters and panel data format, the variables were expressed as follows:

2 $$PM2.5_{it} = \upalpha _{i} +\upbeta _{1} GDP_{it} +\upbeta _{2} COEXP_{it} +\upbeta _{3} FA_{it} +\upbeta _{4} TEMP_{it} +\upbeta _{5} PREC_{it} +\upmu _{it} ,$$

where $\beta_{1} ,\beta_{2} ,\beta_{3} \;{\text{and}}\;\beta_{4}$ are the slope parameters of the explanatory variables and $\alpha_{i} \;{\text{and}}\;\mu_{it}$ are the constant and error terms respectively. Study area and time are symbolized by $i$ and $t$ respectively.

In order to reduce scattering, multi-collinearity and heteroscedasticity, the author transformed all variables into natural logarithms and it is well known that the regression afterward produces more consistent and accurate results than ordinary linear regression^2,53^.

Using Eq. 2, the logarithmic specification can be expressed in the following manner:

3 $$LnPM2.5_{it} = \upalpha _{i} +\upbeta _{1} LnGDP_{it} +\upbeta _{2} LnCOEXP_{it} +\upbeta _{3} LnFA_{it} +\upbeta _{4} LnTEMP_{it} +\upbeta _{5} LnPREC_{it} +\upmu _{it} ,$$

where $i$ denotes the country (i = 1, 2, …, 6), $t$ the time (1998–2020) and $\upmu _{it}$ the error term. The coefficients $\upbeta _{1} ,\upbeta _{2} ,\upbeta _{3} ,\upbeta _{4}$, $\upbeta _{5}$ denote the impacts of the explanatory variables on the responding variable. After log transformation, the variable’s coefficients became elastic.

### Study area

**Figure 2:**
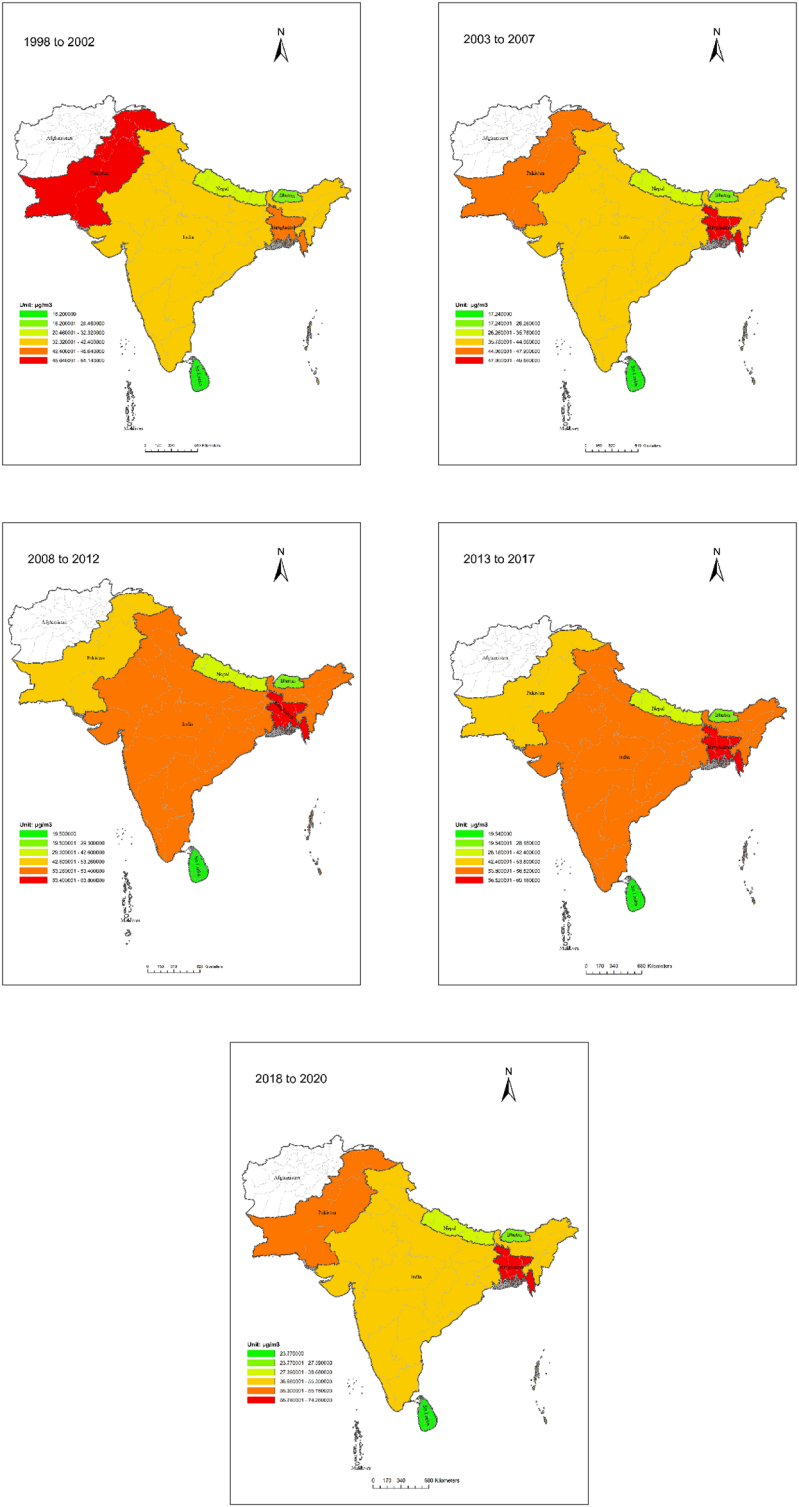
SAARC Region’s PM2.5 concentrations, from 1998 to 2020: A Spatio-temporal distribution. This figure shows the PM2.5 concentration over time and in different regions. The different colors represent the average value of the PM2.5 concentrations of each country, and the color layer was created by ArcGIS10.8. (http://desktop.arcgis.com/en/).

Since it was founded in 1985, the South Asian Association for Regional Cooperation (SAARC) has worked to promote economic, scientific, social and cultural growth in South Asia. Bangladesh, Bhutan, India, Nepal, Pakistan, the Maldives, Afghanistan and Sri Lanka are all part of the group. Afghanistan joined in 2007, making it the most latest country to join the group. The SAARC region was the main topic of this study. The goal was to look at how economic growth, consumer spending, forest area, weather and rainfall might all affect PM2.5 levels. Figure 2 shows data at the country level for a number of factors that help our research in the area. Afghanistan and the Maldives were left out of the study because there wasn’t enough data on them. Panel statistics with real-world information were collected at the country level in the SAARC area from 1998 to 2020.

### Data source and variable selection

Based upon the scattering properties and simulation of aerosol vertical profile^54–56^, this study used a single country-level panel data set or haze pollution proxied by particulate matter 2.5 concentrations and denoted as (PM2.5) (V4.GL.03) for the SAARC region from 1998 to 2020, with a spatial resolution of 10 km^57^. The PM2.5 concentration data were calculated using NASA’s AOD, MODIS and MISR^2^. ACA—Atmospheric Composition Analysis Group has made a version of this data set available online at the group’s website (https://sites.wustl.edu/acag/datasets/surface-pm2-5/). The analysis uses a subset of available data, which covers country-level data for the SAARC region (see Fig. 2). Economic growth was measured as the change in GDP (purchaser’s prices) denoted as GDP, consumer expenditure as final consumption expenditure (constant 2010 US$) denoted as (COEXP) and forest area as the total forest area (sq. km) denoted as (FA). To measure consumers’ expenditure, we used final consumption expenditure (constant 2010 US$) and to indicate the country’s green area we used the total available forest area (sq. km). We created the panel data for economic growth based on the data extracted from the macrotrends database found at: https://www.macrotrends.net/^58^. The World Development Indicators (WDI) (http://data.worldbank.org) data set provided the data for consumer expenditure and forest areas^3^. Climate data such as temperature (TEMP) and precipitation (PREC) were extracted from the WorldClim database (https://www.worldclimate.org/data/monthlywth.html, where TEMP and PREC are measured in degrees Celsius (°C) and millimeters (mm)^59^.

In the context of our research, we have formulated and implemented a system aimed at influencing the determinants of PM2.5 pollution. This system draws upon a comprehensive body of previous studies and incorporates easily accessible and publicly available information. The present study, as depicted in Table 1, investigated a range of influential variables.

**Table 1:** Influencing factor system and the summary of the study’s variables.

Variables	Symbol	Unit	Description	Data source
Particulate matter	PM2.5	µg/m^3^	Indicating the air quality	ACA
Economic growth	GDP	Billion dollar	GDP/capita based on purchaser’s prices	Macrotrends
Consumption expenditure	COEXP	USD $	Final consumption expenditure (constant 2010 US$)	WDI
Forest area	FA	Sq.km	Forest area	WDI
Temperature	TEMP	°C	Average annual temperature	WorldClim
Rainfall	PREC	mm/inch	Total annual precipitation	WorldClim

It explains the variables, the symbol used to illustrate each variable, the unit for each, and the description and the source of the data for each variable.

The response variable of this study is particulate matter and, designated by PM2.5. Meanwhile, the explanatory variables, such as economic growth, consumption expenditure, forest area, annual temperature and annual precipitation, are designated GDP, COEXP, FA, TEMP and PREC, respectively.

### Econometric methods

The issue of cross-sectional dependence (CD) and heterogeneity problems may arise in the context of econometric study using country-level panel data^53,60^. Hence, it is important to do a comprehensive evaluation of the considerations before selecting a suitable analytical methodology to alleviate the occurrence of these challenges. The inclusion of confounding variables CD and heterogeneity in a research might introduce bias and compromise the reliability of the findings^44,60,61^. Consequently, it is advisable to employ “second-generation” econometric approaches where applicable.

#### Cross-sectional dependence test

Previous studies have shown evidence of the presence of cross-sectional dependency (CD) in cases when nations are connected by shared economic and social networks, spatial impacts, or other unobserved variables^62^. The presence of shared shocks has led to the inclusion of CD in the error terms of panel data models^2,60^. Failure to handle this issue can lead to biased and inconsistent results^53,60^. The study incorporates few variants of CD tests, namely: (i) the LM test developed by Breusch and Pagan^63^; (ii) the scaled LM test proposed by Pesaran^44^; (iii) the bias-corrected scaled LM test introduced by Baltagi et al.^64^; and (iv) the CD test developed by Pesaran^44^.

#### Slope homogeneity test

After determining the CD, it is imperative to evaluate the dataset’s homogeneity. To guarantee the dependability of the estimation results, it is critical to confront the concern of cross-sectional heterogeneity, as highlighted by Breitung^61^. The study employed the Pesaran and Yamagata^45^ test to assess the uniformity of slope coefficients. The test in question was chosen by Musah et al.^65^ due to its ability to manage residual cross-sectional dependency effectively and yield reliable and coherent outcomes. The authors, Pesaran and Yamagata^45^ utilized panel data to expand upon the approach developed by Swamy^66^ and confirmed the homogeneity of slopes. This is demonstrated in the following manner:

4 $$\tilde{S} = \sum\limits_{i = 1}^{N - 1} {\left( {\widehat{\beta }_{i} - \tilde{\beta }_{WFE} } \right)^{\prime} } \frac{{X_{i}^{\prime} M_{\tau } X_{i} }}{{\widetilde{\sigma }_{i}^{2} }}\left( {\widehat{\beta }_{i} - \tilde{\beta }_{WFE} } \right),$$

where $\widehat{\beta }$ denotes the coefficients of the pooled OLS for each discrete *i* from *1* to *N* and $\tilde{\beta }_{WFE}$ depicts the weighted fixed effect (WFE) of the slope coefficient’s pooled estimator. Additionally, $M_{\tau }$ depicts the identity matrix and the estimation is the $\widetilde{\sigma }_{i}^{2}$. The $\overline{\Delta }$ is the standardized dispersion statistic and $\overline{\Delta }_{adj}$ is the biased-adjusted version. They are defined by:

5 $$\overline{\Delta } = \sqrt N \left( {\frac{{N^{ - 1} \widetilde{S} - k}}{{\sqrt {2k} }}} \right),$$

6 $$\overline{\Delta }_{adj} = \sqrt N \left( {\frac{{N^{ - 1} \widetilde{S} - E\left( {\overline{z}_{it} } \right)}}{{\sqrt {{\text{var}} \left( {\overline{z}_{it} } \right)} }}} \right),$$

where $$E\left( {\overline{z}_{it} } \right)$$ = $$k$$ and $${\text{var}} \left( {\overline{z}_{it} } \right) = \frac{{2k\left( {T - k - 1} \right)}}{T + 1}$$.

#### Panel unit root test

In panel data analysis, determining the stationarity of variables is crucial for reliable estimations. Panel unit root tests offer a tool for this task, but relying solely on first-generation tests can be treacherous. These tests assume independence across cross-sectional units, an assumption that often crumbles in real-world data. Following Odhiambo^60^, Tugcu^67^ and Jahanger et al.^30^ the twin problem of homogeneity and cross-sectional dependence is reduced in the second-generation panel unit root. They explicitly account for cross-sectional dependence, ensuring robust and accurate assessments of stationarity. This study, recognizing the lurking presence of dependence in its data, wisely wields both first- and second-generation tests. This two-pronged approach ensures that the stationarity conclusions drawn are firmly grounded in fact, not shaky assumptions. The second-generation PANIC and CIPS tests developed by Bai and Ng^46^ and Pesaran^47^, respectively, with their ability to handle various sources of non-stationarity, are particularly valuable additions to the testing arsenal. In the presence of cross-sectional dependence, these tests are the sturdy tools needed to build reliable econometric models. By embracing this methodological rigor, researchers can navigate the complexities of panel data with confidence and draw accurate conclusions about their variables’ stationarity. The equation model for PANIC test is written as.

The date $$X_{it}$$$$(i = 1,......,N;t = 1,....,T)$$ are assumed to be generated by;

7 $$X_{it} = c_{i} + \beta_{i} t + \lambda_{i}^{\prime} F_{t} + e_{it} ,$$

8 $$(I - L)F_{t} = C(L)u_{t} ,$$

9 $$(1 - p_{i} L)e_{it} = D_{i} (l)\varepsilon_{it} ,$$

where $C(L) = \sum\nolimits_{j = 0}^{\infty } {C_{j} L^{j} }$ and $D_{i} L = \sum\nolimits_{j = 0}^{\infty } {D_{ij} L^{j} }$ The idiosyncratic error $e_{it}$ is I (1) if $p_{i} = 1$ and is stationary if $\left[ {p_{i} } \right] < 1$. We allow $r_{0}$ stationary factors and $r_{1}$ common trends, with $r = r_{0} + r_{1}$. Stated differently, the rank of $C(1)$ is $r_{1}$. The objective is to determine $r_{1}$ and test if $p_{i} = 1$ when neither $F_{t}$, nor $e_{it}$ is observed and will be estimated by the method of principal components.

On the other hand, CIPS test is based on the Dickey-Fuller (CADF) test. The equation allows one to difine the CADF that is provided below:

10 $$\Delta y_{it} = \alpha_{i} + \pi_{i} y_{i,t - 1} + \varphi_{i} \overline{y}_{t - 1} + \sum\limits_{l = 0}^{P} {\emptyset_{il} \Delta \overline{y}_{t - 1} } + \sum\limits_{l = 1}^{P} {\gamma_{il} \Delta \overline{y}_{i,t - 1} } + t + \lambda_{i}^{\prime} F_{t} + \in_{it} ,$$

where,$\overline{y}_{t - 1}$ and $\Delta \overline{y}_{i,t - 1}$ are averages for lagged and first differences of each cross-section series. The constant term is denoted by $(i = 1,\dots , t, i),$ the trend by $(t)$, the time lag by $(yi, t j)$ and the error by $\in_{it}$.

A cross-section is used to analyze the results of the CADF test for the whole panel; the section-wise CIPS test might be carried out, as outlined in the following sentence:

11 $$CIPS = \frac{1}{N}\sum\limits_{i = 1}^{N} {CADF_{i} } ,$$

where CADF_i_ represents “cross-sectional augmented dickey fuller test” and N is the number of observations. The covariate-augmented Dickey–Fuller (CADF) test is useful for determining whether or not a series has a unit root that employs a null hypothesis.

#### Panel cointegration test

A cointegration relationship between the variables under consideration must be evaluated once the integration order of the variables is confirmed^2,12^. As a result of cross-sectional dependency, standard cointegration tests, such as Johansen and Kao, might be less accurate, hence leading to inaccurate conclusions. A number of commonly used cointegration tests have been utilized in the study, such as the Pedroni cointegration test^48^ and the Kao Residual cointegration test^49^. On the other hand we also employed the innovative Durbin Hausman group mean cointegration test proposed by Westerlund^50^ to tackle the previously described CD issue. This test holds two notable advantages: firstly, it avoids heavy reliance on prior knowledge regarding the order of integration and relaxes the cross-sectional dependency assumption, allowing for differentiation of explanatory variables based on their stability ranks. Secondly, the Group-mean tests ($G_{\tau }$ and $G_{\alpha }$) scrutinize the cointegration of all units within a group, while the Panel tests ($P_{\tau }$ and $P_{\alpha }$) assess the cointegration of the entire panel^68^. Consequently, the error correction specification for the Westerlund cointegration test^50^ is provided as follows:

12 $$\Delta Y_{i,t} = \delta_{i}^{\prime} d_{t} + \in_{i} \left( {Y_{i,t - 1} - \beta_{i}^{\prime} X_{i,t - 1} } \right) + \sum\limits_{j = 1}^{p} {\varphi_{ij} Y_{i,t - j} } + \sum\limits_{j = 0}^{p} {\varphi_{ij} X_{i,t - j} } + u_{i,t} ,$$

here $\in_{i}$ represents the coefficient’s rate of restitution towards equilibrium.

Here, The four formulations presented by Westerlund^50^, which incorporate group-mean statistics and panel statistics, are shown by Eqs. (13) to (16).

13 $$G_{\tau } = \frac{1}{N}\sum\limits_{i = 1}^{N} {\frac{{ \in_{i} }}{{Se\left( {\widehat{ \in }_{i} } \right)}}} ,$$

14 $$G_{\alpha } = \frac{1}{N}\sum\limits_{i = 1}^{N} {\frac{{T \in_{i} }}{{ \in^{\prime}_{i} \left( 1 \right)}}} ,$$

15 $$P_{\tau } = \frac{{\widehat{ \in }_{i} }}{{Se\left( {\widehat{ \in }_{i} } \right)}},$$

16 $$P_{\alpha } = T\widehat{ \in }.$$

The statistical calculations in the analysis employ the least-squares estimation method for determining the values of variables $\in_{i}$ and $T$. Moreover, the utilization of the $G_{\tau }$ and $G_{\alpha }$ statistics is applied to evaluate the existence of cointegration in at least one cross-sectional unit, while the $P_{\tau }$ and $P_{\alpha }$ statistics demonstrate the presence of cointegration across the complete panel.

#### Empirical model estimation

According to the research conducted by Le and Ozturk^53^ and O’Connell^69^, the existence of cross-sectional dependency (CD) has the potential to invalidate stationarity tests and their corresponding outcomes. The Ordinary Least Squares (OLS) and other renowned econometric approaches have been found to demonstrate bias with the presence of collinearity and heteroscedasticity^53,70^. Furthermore, traditional estimators exhibit a lack of conclusiveness when investigating independent variables as potential factors contributing to cross-sectional dependency. The Pooled Mean Group (PMG) estimation is considered more favorable than the single cointegration technique for a variety of convincing reasons when determining the presence of long- and short-run equilibrium in the provided time series data^71,72^. Sikder et al.^52^ have demonstrated that the PMG approach yields more reliable and efficient results while simultaneously providing both dynamic long- and short-term coefficients^52,73^. As previously mentioned, the existence of CD, SH and long-term relationships among the variables of interest led to the adoption of the second-generation estimation technique as well. The Augmented Mean Group (AMG) model is a sophisticated econometric technique commonly employed for panel data analysis. The AMG model considers the impact of lagged variables to capture the temporal dynamics of individual effects. These models offer a comprehensive set of tools for examining economic phenomena in panel datasets, enabling a detailed examination of individual and collective impacts over time^74^. We have chosen the AMG approach for conducting empirical analysis due to the presence of CS dependence and mixed order of integration in the data. The selected techniques address the issues of CD and heterogeneity, ensuring consistent estimates^74,75^. As a consequence of the aforementioned benefits, we decided to use the PMG and AMG estimator to examine the dynamic associations between the dependent and independent variables.

Based on the PMG framework, we can formulate long-run and short-run models as depicted in Eqs. (17) and (18) below:

17 $$\Delta LnPM2.5_{it} = \alpha_{1} + \sum\limits_{k = 1}^{p} {\beta_{ij} LnPM2.5_{it - j} + \sum\limits_{k = 0}^{q} {\delta_{ij} \Upsilon_{it - j} + \varepsilon_{it} } } .$$

The cross-sectional unit, time frame and optimal lags in Eq. (17), are depicted by $i$, $t$ and $j$ respectively. $\Upsilon_{it}$ indicates the exogenous variables, $\Delta LnGDP$, $\Delta LnCOEXP$, $\Delta LnFA$, $\Delta LnTEMP$ and $\Delta LnPREC$, which are representing economic growth, consumption expenditure, forest area, total temperature and annual rainfall, respectively. Moreover, optimal lag orders are depicted by p and q while $\varepsilon_{it}$ indicated the error terms.

Equation (18) below illustrates the short-run model estimation of the variable studied:

18 $$\Delta LnPM2.5_{it} = \theta_{i} + \sum\limits_{k = 1}^{p} {M_{ij} \Delta LnPM2.5_{it - j} + \sum\limits_{k = 0}^{q} {Z_{ij} \Delta \Upsilon_{it - j} + \phi_{ij} ECT_{t - i} + \varepsilon_{it} } } .$$

The ECT (Error Correction Term) analyzes the selected variables in Eq. (16) over the long term. This analysis establishes the rate at which the long-run equilibrium recovers from short-run disturbances. Following a shock,$ECT_{t - i}$ indicates the direction in which the long-run equilibrium will be converged, as indicated by $\phi_{ij}$. Furthermore, the $ect_{t - 1}$ coefficients has to be statistically significant and negative^52,76^.

The augmented mean group (AMG) approach takes into account cross-sectional dependency by incorporating dynamic effects. Eberhardt and Teal^77^ introduced the AMG approach as an alternative to a two-step method^45^. First, it utilizes ordinary least squares (OLS) in difference form to combine unobserved time dummies as follows in Eqs. (19) and (20):

19 $$\Delta y_{it} = \beta_{o} + \beta_{i} \Delta X_{it} + \beta_{i} \sigma_{t} + \sum\limits_{i = 2} {T\emptyset_{t} D_{t} } + \varepsilon_{it} ,$$

where $y_{it}$ presents dependent variable, $\beta_{i}$ presents explanatory variables, $\Delta$ indicates differenced and $\beta_{o}$ presents intercept. $\beta_{i}$, $\sigma_{t}$ and $\emptyset_{t}$ indicates the slope of cross-sections, unobserved common effects and heterogenous factors. Parameters for AMG model for every cross-section are averaged as:

20 $$AMG = \frac{1}{N}\sum\limits_{i = 1}^{N} {\overline{\beta }_{i}^{{}} } .$$

Scientists use two-stage least squares (TSLS) with instrumental variables when regular linear regression methods like OLS aren’t suitable. This is especially helpful when independent variables are correlated with the error term^78,79^. TSLS coefficients are generally unbiased in large samples, unlike OLS coefficients. Modern software calculates TSLS coefficients directly. The utilization of the Two-Stage Least Squares (2SLS) method might present difficulties due to the necessity of specifying a set of instruments that are known a priori to be uncorrelated with the error term. In linear simultaneous equation models, exogenous variables (unaffected by the model) can serve as instruments. A variable (X and Z) can be both explanatory and an instrument if it's not correlated with the error term. The estimation of the coefficients of the linear model is given below:

21 $$Y_{i} = \beta_{0} + \beta_{1} X_{1i} + \cdots + \beta_{p} X_{pi} + \varepsilon_{i} .$$

When it is possible to find a correlation between the error term and the endogenous variable indicated by $X_{ji}$ as has been previously mentioned in a number of academic publications. The ordinary least squares (OLS) estimate of the equation in question would exhibit bias and inconsistency under the given circumstances. The steps involved in estimating $\beta$ utilizing a two-stage least squares technique are as follows:

1. Save the predicted values $\hat{X}_{j}$ after regressing each $X_{j}$ on $Z$. Then we will have $\hat{X}_{j} = X_{j}$ if $X_{j}$ is included in $Z$.
2. Analyze the regression model using OLS estimation to determine $\beta$.
  22 $$Y_{i} = \beta_{0} + \beta_{1} \hat{X}_{1i} + \cdots + \beta_{p} \hat{X}_{pi} + \varepsilon_{i} .$$

Generalized least squares (GLS) addresses heteroscedasticity, cross-sectional dependence and serial correlations in panel regression models by incorporating them into the estimation process. It’s a comprehensive solution for these issues. FGLS, a statistically effective estimator for systems with both serial and contemporaneous correlations, was introduced by Parks^80^. Bai et al.^81^ proposed a new method for estimating the error covariance matrix in high-dimensional settings, building upon FGLS to reduce biases from cross-sectional and serial correlation. Their approach is effective even without cluster information. Consider a stochastic sample of $n$ observations from a data-generating process;

23 $$Y = X\beta + \mu ,$$

where $Y$ and $\mu$ are vectors of outcomes and errors, $X$ is an $n$ by $p$ matrix of strictly exogenous regressors and $\beta$ is a p-vector of parameters. The diagonal matrix $W = E\left[ {uu^{\prime}|X} \right]$ has $i$th diagonal element $w_{ii}$, which is an unknown function of Xi $X_{i}$ alone: $w_{ii} = {\text{var}} \left( {\mu_{i} |X_{i} } \right)$.

The FGLS estimator;

24 $$\hat{\beta }_{FGLS} = [X^{\prime}\hat{\Omega }^{ - 1} X]^{ - 1} X^{\prime}\hat{\Omega }^{ - 1} Y,$$

of Eq. (23) uses an estimate $\hat{\Omega }$ of $\Omega$ with entries $\hat{\Omega }_{ii}$. We take the common approach of setting $\hat{\Omega }_{ii} = \exp \left( {\hat{g}\left( {X_{i} } \right)} \right)$ for a function $\hat{g}$ estimated using the log of squared residuals $\log \left( {\hat{u}_{i}^{2} } \right)$ from initial OLS estimation of (23).

#### Causality test for panel data

In this investigation, the Dumitrescu and Hurlin causality test, a widely employed panel variant of the Granger causality test Granger^82^, was utilized. This test is founded on the non-causality hypothesis put out by Dumitrescu and Hurlin^51^. Granger’s non-causality approach recognizes the significance of considering heterogeneity in panel data, as highlighted by Ji and Yang^83^ and Jahanger et al.^30^. This test is a versatile D–H test that can be applied to a wide range of data, including diverse and imbalanced datasets, similar to the TN or T > N. To evaluate the causal relationships between the variables, the study utilized the D–H test. In contrast to the H1 hypothesis, the H0 hypothesis of this assessment argues that there is no causal relationship between the parameters. However, the evaluation presents an opposing H1 hypothesis, suggesting that there is a causal connection based on findings from at least one cross-sectional study. Equation (25) is a valuable tool that utilizes the D-H diagram. The D-H causality test is expressed in Eq. (25), where Y represents the dependent variable and X represents the independent variable. It can be written as follows:

25 $$y_{it} = \varphi_{i} + \sum\limits_{k = 1}^{P} {\gamma_{i}^{k} y_{i,t - k} } + \sum\limits_{k = 1}^{P} {x_{ik}^{j} T_{i,t - k} } + \omega_{i,t} .$$

Here, the autoregressive parameters are represented by $zi (j)$, where as k stands for the lag length. Specifically, the slope coefficients are $i$ and $i$, the slope-intercept is $i$ and the error term is $t$.

## Empirical results and discussion

### Results of descriptive statistics and correlation analysis

The tabulated descriptive statistics and correlation analysis in Table 2 reveal that the mean values of LnCOEXP, LnFA and LnPREC exceed those of the other variables. Concurrently, the cumulative data portrays a positive trend, supported by LnPM2.5, LnGDP, LnCOEXP, LnFA, LnPREC and LnTEMP.

**Table 2:** The descriptive statistics and correlation coefficients of variables.

Statistics	LnPM2.5	LnGDP	LnCOEXP	LnFA	LnPREC	LnTEMP
Descriptive statistics						
Mean	3.62	3.88	24.51	10.85	5.50	2.38
Median	3.74	4.20	24.84	10.37	5.52	2.08
Maximum	4.38	7.96	28.32	13.49	6.39	3.25
Minimum	2.67	− 1.02	19.97	9.84	4.14	1.80
Std. Dev.	0.44	2.27	2.21	1.23	0.62	0.56
Skewness	− 0.49	− 0.29	− 0.40	1.41	− 0.37	0.66
Kurtosis	2.15	2.45	2.52	3.50	2.09	1.52
Jarque–Bera	9.64	3.70	4.95	47.46	7.95	22.59
Probability	0.0080	0.1570	0.0839	0.0000	0.0187	1.5225
Normality	The series is distributed normally					
Correlation analysis						
LnPM2.5	1					
LnGDP	0.60	1				
LnCOEXP	0.57	0.99	1			
LnFA	0.34	0.58	0.60	1		
LnPREC	− 0.32	0.24	0.24	0.31	1	
LnTEMP	− 0.16	0.15	0.15	− 0.45	0.54	1

The table illustrates the result of descriptive statistics and the correlation between variables where data is comprehensively explanatory.

All variable distributions exhibit leptokurtic characteristics, indicated by a positive Kurtosis. The normality of these distributions was assessed through the Jarque–Bera test, which confirmed their adherence to a normal distribution. Furthermore, the variables displayed significant correlations, as demonstrated in Table 1, with all panels showing robust associations with PM2.5 pollution.

### Results cross-sectional dependence tests

Numerous tests were utilized to assess cross-sectional dependence, such as the Pesaran CD test, the Breusch-Pagan LM test, the Pesaran scaled LM test and the Bias-corrected scaled LM test. Prior research has employed and demonstrated these tests in the different studies^2,45^. The findings of the examinations, which are presented in Table 3, unequivocally reject the null hypothesis that no cross-sectional dependence exists among the countries comprising the panel. 1%, 5% and 10% significance levels provide additional support for this rejection. Therefore, the results of this research indicate that the countries under investigation demonstrate cross-sectional interdependence, underscoring the possibility that an issue or disruption in one nation could spread and affect others.

**Table 3:** Cross-sectional dependence results of the variable studied.

Series	Cross-sectional dependence results			
	Breusch–Pagan LM	Pesaran scaled LM	Bias-corrected scaled LM	Pesaran CD
LnPM2.5	160.7248*** (0.0000)	26.60559*** (0.0000)	26.46923*** (0.0000)	12.24260*** (0.0000)
LnGDP	329.5897*** (0.0000)	57.43596*** (0.0000)	57.29960*** (0.0000)	18.15187*** (0.0000)
LnCOEXP	337.7183*** (0.0000)	58.92002*** (0.0000)	58.78366*** (0.0000)	18.37676* (0.0000)
LnFA	304.1627** (0.0000)	52.79364** (0.0000)	52.65727*** (0.0000)	− 3.645848* (0.0003)
LnPREC	47.50281** (0.0000)	5.934175*** (0.0000)	5.797811*** (0.0000)	3.828086** (0.0001)
LnTEMP	97.85076*** (0.0000)	15.12641*** (0.0000)	14.99005*** (0.0000)	7.570104*** (0.0000)

***, ** and * indicate the statistical significance of 1%, 5% and 10% respectively. All variables are significant at 1%, 5% and 10% level, denoted by ***, ** and *

### Results of panel slope homogeneity test

Le and Ozturk^53^ highlight the need of examining the diversity of slope coefficients using the Pesaran and Yamagata^45^ test. The data exhibits slope variability, as seen in Table 4. The null hypothesis of slope homogeneity is significantly rejected at both the 1% and 5% levels, indicating considerable variability in parameters between nations.

**Table 4:** Results of the slope heterogeneity test.

Statistics	Value	Probability
Delta ($\tilde{\Delta }$)	2.143	0.032**
Adj. ($\tilde{\Delta }_{adj}$)	− 2.203	0.010***

***, ** and * indicate the statistical significance of 1%, 5% and 10% respectively.

### Results of panel unit root test

The assessment of stationarity has significant importance in ascertaining the integration characteristics of the variables^2^. In order to assess the existence of cross-sectional dependency and heterogeneity in slopes, we employed the first and second-generation unit root tests, as presented in Tables 4 and 5. These tests were applied to ascertain the suitable integration order of the variables for further econometric analyses. Table 5 presents the results of the Levin Lin Chu t-statistics, the IPS W-statistics, the Fisher ADF Chi-square and the Fisher PP Chi-square unit root tests. In addition, the analysis of the integration of variables’ sequencing involved the utilization of second-generation unit root tests, namely PANIC developed by Bai and Ng^46^ and CIPS developed by Pesaran^47^. The tests in question are highly acknowledged for their capacity to successfully tackle residual cross-sectional dependency.

**Table 5:** Result of the first-generation panel unit root tests.

Variables	LLC t- statistics		IPS W- statistics		Fisher ADF Chi-square		Fisher PP Chi-square	
	Level	1st diff.	Level	1st diff	Level	1st diff.	Level	1st diff.
LnPM2.5								
Intercept	− 3.16923***	− 12.4995***	− 2.28773**	− 11.6101***	24.2489**	110.679***	25.1522**	210.432***
Trend	− 2.88433***	− 8.90333***	− 2.51376***	− 9.78607***	24.9036**	85.2774***	24.0390**	443.865***
LnGDP								
Intercept	− 0.86102	− 5.16713***	0.98737	− 4.15321***	14.0331	40.2673***	8.27277	43.9665***
Trend	1.05962	− 4.54236***	3.69778	− 2.89579**	4.42235	28.6548***	7.78101	40.5816***
LnCOEXP								
Intercept	− 0.43602	− 1.52891*	3.03555	− 5.44393***	1.77968	51.8032***	1.62552	124.697***
Trend	− 1.68562**	0.26800	− 3.31189***	− 3.50696***	35.1946***	34.8415***	27.0848***	102.870***
LnFA								
Intercept	− 1.03509	− 6.44272***	1.35251	− 6.22732***	5.79152	57.6627***	12.3976	58.4613***
Trend	− 0.13529	− 3.38380***	0.69755	− 4.12020***	7.35033	36.4409***	9.84137	40.7081***
LnPREC								
Intercept	− 9.05127***	− 8.59535***	− 7.52378***	− 12.9819***	70.7320***	126.302***	72.9885***	796.234***
Trend	− 7.52068***	− 6.08025***	− 7.16378***	− 11.3895***	62.1761***	99.7363***	196.503***	867.235***
LnTemp								
Intercept	− 6.63637***	− 13.7248***	− 5.57900***	− 12.9832***	51.6274***	128.313***	51.8093***	679.006***
Trend	− 7.07326***	− 11.4948***	− 5.48650***	− 11.4181***	49.3866***	101.023***	49.8957***	609.863***

***, ** and * indicate the statistical significance of 1%, 5% and 10% respectively.

The outcomes of the preliminary unit root tests, as presented in Table 5, suggest that the variables demonstrate first-order integration, commonly referred to as I(1). The findings of the unit root testing conducted on the second-generation data, as displayed in Table 6, offer additional substantiation for the conclusions derived from the first tests conducted on the first-generation data. The findings of the Bai and Ng’s PANIC test are presented in Table 6. The results suggest that, except for LnPREC, which displays stationarity at the level with both a constant and a trend, the other five variables show stationarity at the first difference, indicated as I (1), either with a constant or with a trend. Based on the empirical results obtained from the Pesaran’s CIPS test, it is evident that all variables exhibit stationarity at the first difference when a constant term is incorporated. Hence, irrespective of whether first-generation or second-generation unit root tests are employed, it can be conclusively established that all variables attain stationarity by initial differentiating.

**Table 6:** Results of the second-generation unit root tests.

Variables	Bai and Ng (PANIC)				Pesaran’s CIPS			
	Level		1st diff.		Level		1st diff.	
	Constant	Trend	Constant	Trend	Constant	Trend	Constant	Trend
LnPM2.5	− 0.5218	− 1.81151**	− 1.77661*	− 1.23349	− 2.26238*	− 2.76935*	− 5.62226***	− 5.61366***
LnGDP	− 0.18784	0.65989	2.59557***	4.55044***	− 2.46846**	− 2.68410	− 3.33366***	− 3.01742**
LnCOEXP	− 0.08327	− 1.51129	3.78674***	− 0.86414	− 2.79787***	− 2.51024	− 2.89314***	− 2.58629
LnFA	2.59482***	− 0.41366	− 2.44704**	− 2.36222**	− 2.84730***	− 4.34489***	− 5.37697***	− 4.80644***
LnPREC	7.39105***	9.37742***	0.37701	1.18911	− 3.46115***	− 3.21770***	− 5.69754***	− 5.15974***
LnTEMP	5.06102***	0.20967	6.61595***	9.17195***	− 2.67818***	− 4.25902***	− 6.81446***	− 6.25701***

***, ** and * indicate the statistical significance of 1%, 5% and 10% respectively.

### Results of panel cointegration test

Further investigation into the cointegration relationship between the carefully selected variables is imperative, building upon the research presented in the literature^2^. The primary objective of our research is to ascertain the potential existence of a long-term equilibrium phenomenon. To accomplish our aim, we have employed the dependable cointegration tests that Westerlund^50^, Pedroni^48^ and Kao^49^ have suggested. The efficacy of these tests in detecting enduring relationships between variables has been thoroughly verified, with consideration given to the well-documented presence of cross-sectional dependence, slope heterogeneity and order of integration.

The results shown in Table 7 demonstrate a persistent and consistent relationship between the variables. According to the results obtained from the Westerlund test, a statistically significant and negative long-term association has been observed between the variables of interest, as indicated by the $Gt$ and $Pt$ tests. The Redundant Fixed Effect—Likelihood Ratio Test results indicate that the Ordinary Least Squares (OLS) fixed effect model exhibits statistical significance at the 1% level.

**Table 7:** Results of panel cointegration test.

		Statistics	Probability
Pedroni^48^ test for cointegration			
Panel cointegration or within-dimension statistics			
Modified variance ratio		− 3.1723***	0.0008
Modified Phillips–Perron t		1.8550**	0.0318
Phillips–Perron t		− 1.3084*	0.0954
Augmented Dickey–Fuller t		− 1.2601	0.1038
Group-mean or between-dimension statistics			
Modified Phillips–Perron t		2.6259**	0.0043
Phillips–Perron t		− 1.3115*	0.0949
Augmented Dickey–Fuller t		− 1.6230*	0.0523
Kao^49^ test for cointegration			
Modified Dickey–Fuller		− 2.7643**	0.0029
Dickey–Fuller		− 3.0929***	0.0010
Augmented Dickey–Fuller		− 1.5128	0.0652
Unadjusted modified Dickey–Fuller		− 7.4424***	0.0000
Unadjusted Dickey–Fuller		− 4.6646***	0.0000

Statistics	Value	Z-value	Robust p-value
Westerlund^50^ test for cointegration			
$Gt$	− 2.844**	− 0.509	0.050
$Ga$	− 6.626	2.481	0.250
$Pt$	− 7.010**	− 1.107	0.030
$Pa$	− 7.066	1.249	0.120

Statistics			Probability
Redundant fixed effect—likelihood ratio test			
66.2211***			0.0000

***, ** and * indicate the statistical significance of 1%, 5% and 10% respectively.

### Estimations of models

#### PMG and AMG estimation for the dynamic long-run and short-run relationships

In light of the panel’s dependence and heterogeneity, it was desirable to use regression estimators capable of accounting for the issues mentioned earlier throughout the elasticity estimation phase. This PMG estimators was considered suitable for robust regression in our research based on the work of Refs.^65,84–88^. It was used to test the magnitude and direction of various factors affecting PM2.5 concentrations in this region. Since there is the existence of CD and SH, we also have employed the second-generation panel data model in this study to estimate long run and short run dynamics, drawing from various relevant sources^74,75^. Thus, the AMG a second-generation panel data model used in this study since it takes into account cross-sectional dependency by incorporating dynamic effects. As PMG result shown in Table 8, all variables, except for forest area and average temperature, exhibit statistical significance at the 1% significance level. PM2.5 pollution is positively impacted by economic growth and forest area over the long term. Alternatively, final consumption expenditure, total rainfall and average temperature have a negative impact. It is statistically estimated that a 1% increase in LnGDP and LnFA would result in an increase in PM2.5 pollution by 0.48% (− 0.00047%) and 0.12% (− 2.49%) over the long run (short-run), respectively. In contrast, a 1% increase in LnCOEXP, LnPREC and LnTEMP results in a − 0.55% (− 0.50%), − 0.63% (− 0.015%) and − 0.19% (− 0.36%) decrease in PM2.5 pollution over the long-run (short-run). At a 1% error correction level, the error correction term (ECT) was significantly negative (− 0.55).

**Table 8:** The results of dynamic long-run and short-run relationships (PMG and AMG).

Variables	PMG		AMG	
	Coefficient	$p$-value	Coefficient	$p$-value
Long-run estimation				
LnGDP	0.488***	0.000	− 0.076	0.523
LnCOEXP	− 0.550***	0.000	− 0.039	0.777
LnFA	0.120	0.763	− 2.407	0.150
LnPREC	− 0.627***	0.0001	− 0.173*	0.050
LnTEMP	− 0.188	0.636	− 0.765	0.466
Short-run estimation				
COINTEQ01	− 0.551***	0.000	− 0.974***	0.000
D(LnGDP)	− 0.000469	0.996	− 0.177	0.291
D(LnCOEXP)	− 0.496	0.248	− 0.636*	0.054
D(LnFA)	− 2.486	0.635	− 0.743	0.472
D(LnPREC)	− 0.016	0.781	− 0.116	0.214
D(LnTEMP)	− 0.356	0.423	− 0.177	0.291
C	9.819***	0.000	0.023	0.306

***, ** and * indicate the statistical significance of 1%, 5% and 10% respectively.

On the other hand, the AMG result shows that all variables are negatively associated with haze pollution in the long run and the only significant relationship among them is between annual rainfall and haze at a 10% level. In the short run, we can see similar negative associations between all variables and haze, but this time, the significant relationship between consumer expenditure and haze is at a 10% level. It clearly shows that consumer expenditure reduces haze pollution. Statistically, a 1% increase in LnGDP, LnCOEXP, LnFA, LnPREC and LnTEMP would result in a decrease in haze pollution by 0.076%, 0.039%, − 2.407%, 0.173% and − 0.765 in the long run and (− 0.177%), (− 0.636%), (− 0.743%), (− 0.116%), (− 0.177%) in the short run. At a 1% error correction level, the error correction term (ECT) was significantly negative (− 0.974).

#### Robustness checks

Using the approaches of Bai et al.^81^, Yan et al.^89^ and Angrist and Pischke^79^ the TSLS estimations and the FGLS estimations were used to verify the robustness of the dynamic long-term PMG results (see Table 9). The study confirms that the PMG and AMG techniques are suitable for assessing the relationship between economic growth, forest area, average temperature, final consumption expenditure, total rainfall and PM2.5 pollution in SAARC countries. The panel estimates obtained by the Two-Stage Least Squares (TSLS) and Feasible Generalized Least Squares (FGLS) methods reveal a persistent positive relationship between economic growth, forest area and average temperature over time. Additionally, a statistically significant negative association is shown between final consumer expenditure and total rainfall with respect to PM2.5 pollution levels.

**Table 9:** Results of the panel TSLS and FGLS robustness approach.

Variables	Panel TSLS	Panel FGLS
LnGDP	0.505166*** (0.056)	0.3155619*** (0.050)
LnCOEXP	− 0.568513*** (0.064)	− 0.2208492*** (0.056)
LnFA	0.593810*** (0.065)	0.096266** (0.043)
LnPREC	− 1.115529*** (0.092)	− 0.03758907*** (0.059)
LnTEMP	1.140958*** (0.132)	0.1295727 (0.087)
C	12.56698*** (1.201)	8.515427*** (1.054)

***, ** and * indicate the statistical significance of 1%, 5% and 10% respectively.

### Results of panel causality tests

**Figure 3:**
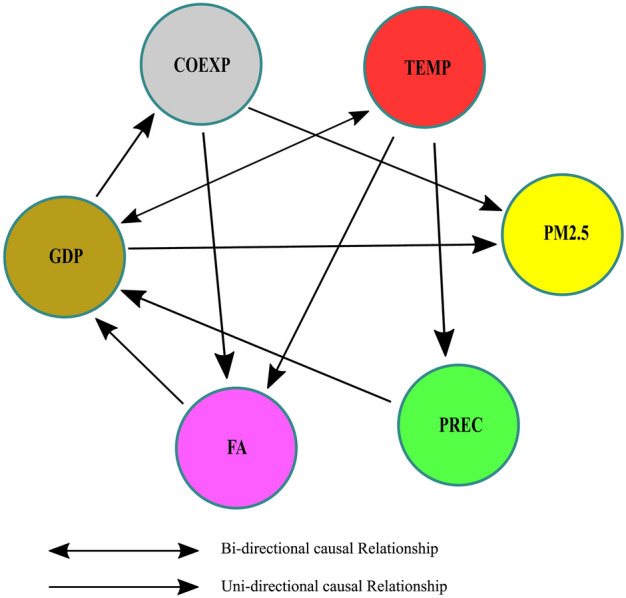
Causal directions between the dependent variable PM2.5 and the independent variables GDP, COEXP, FA, PREC and TEMP. This graphical representation of the causal relations explains the bi-directional and uni-directional causal effect between dependent and independent variables. The (↔) signifies two-way or bi-directional causality and the (→) represents one-way or uni-directional causality between variables.

After assessing the robustness and long- and short-term relationships of the coefficients in the previous section, authors goal in this section of the study is to look into the causative directions among the variables. Using the D–H panel causality test, which was introduced by Dumitrescu and Hurlin^51^, to ensure robustness instead of the traditional Granger^82^ causality test because it has been shown to be a more accurate and efficient method^52^. Due to its ability to differentiate between two forms of heterogeneity inside regression models and homogeneity within random associations^90^, the approach has been utilized by several academics in recent years^52,91^. Table 10 and Fig. 3 show the Dumitrescu and Hurlin panel causality test results and the graphical representation and confirms the strong causal relationships; eight of them are unidirectional and one is bidirectional. The causality analysis demonstrates that there is evidence of feedback effects in the correlations between PM2.5 concentration and other parameters.

**Table 10:** Dumitrescu and Hurlin panel causality results.

Null hypothesis	W-Stat.	Zbar-Stat.	P-value	Result
LnGDP $\Rightarrow$ LnPM2.5	4.2325	1.8068	0.0708*	Uni-directional
LnPM2.5 $\Rightarrow$ LnGDP	3.8883	1.4873	0.1369	No causality
LnCOEXP $\Rightarrow$ LnPM2.5	4.6381	2.1832	0.0290**	Unidirectional
LnPM2.5 $\Rightarrow$ LnCOEXP	2.5702	0.2640	0.7917	No causality
LnFA $\Rightarrow$ LnPM2.5	3.4951	1.1224	0.2617	No causality
LnPM2.5 $\Rightarrow$ LnFA	1.3055	− 0.9096	0.3630	No causality
LnPREC $\Rightarrow$ LnPM2.5	2.9587	0.6246	0.5322	No causality
LnPM2.5 $\Rightarrow$ LnPREC	2.9536	0.6198	0.5353	No causality
LnTEMP $\Rightarrow$ LnPM2.5	3.8879	1.4869	0.1370	No causality
LnPM2.5 $\Rightarrow$ LnTEMP	2.2875	0.0017	0.9986	No causality
LnCOEXP $\Rightarrow$ LnGDP	3.1274	0.7812	0.4347	No causality
LnGDP $\Rightarrow$ LnCOEXP	4.7630	2.2991	0.0215**	Uni-directional
LnFA $\Rightarrow$ LnGDP	5.2699	2.7695	0.0056***	Uni-directional
LnGDP $\Rightarrow$ LnFA	1.5212	− 0.7094	0.4780	No causality
LnPREC $\Rightarrow$ LnGDP	4.6084	2.1556	0.0311**	Uni-directional
LnGDP $\Rightarrow$ LnPREC	2.4035	0.1093	0.9129	No causality
LnTEMP $\Rightarrow$ LnGDP	6.2294	3.6601	0.0003***	Bidirectional
LnGDP $\Rightarrow$ LnTEMP	4.0702	1.6561	0.0977*	Bidirectional
LnFA $\Rightarrow$ LnCOEXP	8.2013	5.4902	4.E−08	No causality
LnCOEXP $\Rightarrow$ LnFA	4.2692	1.8408	0.0656*	Uni-directional
LnPREC $\Rightarrow$ LnCOEXP	0.8758	− 1.3084	0.2840	No causality
LnCOEXP $\Rightarrow$ LnPREC	3.2146	0.8620	0.3886	No causality
LnTEMP $\Rightarrow$ LnCOEXP	1.1312	− 1.0714	0.2840	No causality
LnCOEXP $\Rightarrow$ LnTEMP	2.46878	0.1699	0.8651	No causality
LnPREC $\Rightarrow$ LnFA	2.8489	0.5227	0.6012	No causality
LnFA $\Rightarrow$ LnPREC	3.2243	0.8711	0.3837	No causality
LnTEMP $\Rightarrow$ LnFA	0.3665	− 1.7811	0.0749*	Uni-directional
LnFA $\Rightarrow$ LnTEMP	1.9939	− 0.2708	0.7865	No causality
LnTEMP $\Rightarrow$ LnPREC	4.3619	1.9268	0.0540*	Uni-directional
LnPREC $\Rightarrow$ LnTEMP	3.02117	0.6825	0.4949	No causality

***, ** and * indicate the statistical significance of 1%, 5% and 10% respectively.

Our findings suggest a uni-directional causal relationship moving from LnGDP, LnCOEXP to LnPM2.5, LnGDP to LnCOEXP, LnFA to LnGDP, LnPREC to LnGDP, LnCOEXP to LnFA, LnTEMP to LnFA and LnTEMP to LnPREC in the SAARC countries. This suggests that there is a relationship between economic development and consumption expenditure, both of which have an impact on PM2.5 pollution. Additionally, economic growth is found to influence consumption expenditure. Moreover, the presence of forest area and annual rainfall in a given region significantly affects economic growth. Furthermore, average temperature plays a role in determining the total rainfall in all emerging economies. Moreover, a reciprocal causal relationship exists between the logarithm of temperature (LnTEMP) and the logarithm of gross domestic product (LnGDP). The yearly temperature has a crucial role in the progression of economic growth. Similarly, the yearly temperature of nations belonging to the South Asian Association for Regional Cooperation (SAARC) is significantly influenced by economic development, particularly in instances when there is a notable increase in gross domestic product (GDP). The interrelation of the variables implies that addressing pollution requires the adoption of cross-sectional efforts.

There are a number of regional studies that are consistent with these findings, including studies on the SAARC^2^, MENA countries^92^, BRICS^52^, Central Asia^72^, South East Asia^93^, East Asia^94^, Beijing–Tianjin–Hebei region^8^, sub-Saharan African^60^ Gulf Cooperation Council countries^95^ and other Asian economies^96^. According to our findings, economic growth patterns should be transformed in order to balance the emission reduction policy through more eco-friendly economic activities and the use of green technologies in economic development. The region is experiencing strong economic expansion, which has led to a progressive acceleration in final consumer expenditure, temperature, industrialization, energy usage and urbanization. However, this growth has also resulted in a decrease in forest area and rainfall. The per capita consumption expenditures of residents serve as an indicator of their consumption situations. Previous research have indicated that cities with higher consumption levels tend to exhibit elevated PM2.5 concentrations. This can be attributed to the presence of a greater number of houses, autos, industries and domestic appliances inside these urban areas^8,97^. Studies by Dasgupta et al.^98^ and Wu et al.^8^ indicate that increasing income levels can result in an increase in consumption expenditure level, which may lead to increased industrialization and urbanization, which may adversely affect the environment, particularly in the primary stages of economic growth trajectory in developing countries.

The acceleration of economic growth in emerging nations has been attributed to several factors, including income, consumption spending, industrialization and urbanization^2^. Therefore, a rise in personal income results in a corresponding rise in consumer expenditure, whereas higher rates of urbanization are associated with an escalation in PM2.5 pollution levels. The ongoing pursuit of income development and employment opportunities in developing economies has led to a rise in final consumer spending. However, this has also brought about a dual impact of economic expansion and industrialization, which tends to overshadow the positive externalities linked to environmental degradation. The outcome of this particular circumstance entails a rise in energy consumption, subsequently resulting in heightened degrees of environmental degradation at both the national and regional scales. Furthermore, the escalation of family earnings has an impact on consumer demand, resulting in a consequential expansion of the manufacturing sector that propels industrial development. Consequently, this phenomenon engenders elevated levels of pollution. To avoid such a scenario and reduce anticipated emission levels, Adeneye et al.^99^ and Sikder et al.^52^ suggested adopting renewable energy alternatives in a greater amount. Mahmood et al.^100^, in their latest study conducted on Saudi Arabia, has substantiated the pressing necessity for implementing more stringent urban and industrial-environmental rules in order to curb the escalating levels of pollution. There is a direct relationship between the amount of forest cover, its ecosystem^101^ and the city’s ecological environment. Ji et al.^34^ found that the process of plant absorption has the potential to substantially decrease atmospheric particulate matter, leading to a notable enhancement in air quality through the considerable reduction of particle matter. The adsorption capacity of particulate matter is positively correlated with an increase in forest cover. Lin et al.^102^ conducted a study in an urban setting characterized by expansive forested regions and saw a notable decrease in PM2.5 concentrations. Furthermore, the researchers identified a considerable spillover impact in this context. Additionally, they concluded that the expansion of urban forests contributes to the reduction of PM2.5 levels at local, regional and national levels. Several studies in natural science have demonstrated that green barriers and urban vegetation can reduce pollution concentrations on the street^103^ while having a modest effect on particle deposition on the regional scale^104^.

Moreover, upon scrutinizing the short-run and long-run elasticities presented in Table 7, it is evident from PMG estimation that all variables, except LnFA, exhibit long-term significance in relation to PM2.5 pollution. Nevertheless, none of the variables exhibit a statistically significant correlation with PM2.5 pollution in the short term. Therefore, in contrast to other long-term factors such as electricity generation, transportation system operation and industry expansion, a substantial proportion of PM2.5 emissions from this area can be attributed to economic growth. Furthermore, there exists a correlation between PM2.5 levels, economic development and consumption expenditure that is not statistically significant. On the other hand, the AMG estimator depicts the negative correlation between haze and other variables in the long and short run. This means, in both cases, the emerging economies in the SAARC region will be able to help reduce pollution. The absence of substantial results could potentially be ascribed to the impact of various factors during the short-term estimation period, including increasing income levels, diminishing forest area, decreased total precipitation and elevated average temperatures. This observation suggests that the aforementioned variables do not have a substantial short-term impact on PM2.5 pollution in the emerging economies of this region. There would be many factors that could have caused this to happen. Usage of green technology in industrial production, more green areas, modern high-tech technology usage and the early adoption of environmental conservation laws and policy implementation.

Nevertheless, its significance escalates with the progression of economic and industrial development. A significant coefficient for the lagged error correction term (ECT), $\alpha$[CointEq (− 1)] for PMG and AMG, signifies the existence of a long-lasting relationship between the dependent and independent variables. Therefore, the annual rate of 0.55% and 0.97% for PMG and AMG governs the fluctuations in PM2.5 concentration levels that deviate from short-term to long-term equilibrium, as indicated by the ECT-1 coefficient of − 0.551 and − 0.974.

The findings of the D–H panel causality test in this study provide evidence supporting the presence of a comparable causal association among the various indicators in both Central Asia and Southeast Asian nations. These findings align with several recent studies conducted by Arshad et al.^90^, Shafique et al.^105^ and Hashmi et al.^106^. Furthermore, Saud et al.^107^ and Saidi and Hammami^108^ have identified a causal association between environmental deterioration and GDP growth in countries participating in the Belt and Road Initiative (BRI). In addition, according to Chandran and Tang^109^, Afridi et al.^110^ and Islam^111^, here exists a reciprocal relationship between per capita income and environmental degradation in Indonesia, Thailand, MENA and SAARC nations. The findings of our research align with the conclusions drawn by Chandran and Tang^109^, herein they observed a unidirectional causal relationship between GDP development and environmental deterioration in Malaysia. The results described in the present study, however, are in contrast to those reported in previous investigations. The studies conducted by Asumadu-Sarkodie and Owusu^112^ provided evidence supporting the existence of causal relationships between PM2.5 concentration and energy consumption. Furthermore, these studies also indicate that industrialization is causally linked to both PM2.5 concentration and energy consumption, while economic growth is associated with PM2.5 concentration. According to Sahoo and Sethi^113^, posit that there exists a unidirectional causal link between industrial expansion and energy consumption. Consequently, this study presents a distinct set of results that suggest the absence of a bidirectional or unidirectional causal association between forest area, total rainfall and average temperature within SAARC countries, in contrast to the findings reported by Musa et al.^2^ and Hashmi et al.^106^.

In brief, the results of the PMG analysis validate the existence of a statistically significant and positive correlation and the AMG depicts a negative correlation between economic growth and the concentration of PM2.5 in the SAARC region. Our findings on the impact of economic growth (LnGDP) on PM2.5 concentration are consistent with existing research by Musa et al.^2^ and Sultana et al.^114^, they also report a positive and a negative correlation between this factors. This suggests that economic development in SAARC countries is accompanied by increased air pollution and which is also well marched with revered U-shaped EKC theory. On the contrary, in both estimations, there is a significant negative correlation between annual precipitation and PM 2.5 concentration and both consumer spending and annual expenditures persist over an extended period of time. The negative correlation between rainfall (LnPREC) and PM2.5 observed in our study aligns with findings by Musa et al.^2^ who demonstrate the cleansing effect of precipitation on air pollution. It is critical to observe, nevertheless, that economic expansion has a dual impact. One aspect to consider is its contribution to the increase in PM2.5 concentrations. Nonetheless, it generates increased income levels, which in turn stimulates industrialization, urbanization, consumer spending and energy usage. As a result, this procedure ultimately contributes to the degradation of the environment confirmed by Dasgupta et al.^98^ and Wu et al.^8^. Furthermore, we have effectively established a causal relationship in both directions between temperature and economic growth, which in turn results in a unidirectional causal relationship with PM2.5 concentration. Moreover, a unidirectional causal relationship between economic growth, consumer expenditure and PM2.5 concentration has been identified. Likewise, temperature and consumer spending influence forest area, which subsequently effects economic expansion. Furthermore, annual precipitation has an impact on economic expansion, which in turn effects the concentration of PM2.5. Drawing from the results obtained from our research, it is advisable that emerging economies, such as those that are part of the South Asian Association for Regional Cooperation (SAARC), contemplate incorporating cutting-edge technologies and scientific progress into their strategies to alleviate pollution in the long run. The study’s substantial findings may offer significant insights into socio-economic aspects and dynamic strategies for pollution reduction when developing regional policy initiatives. While economic expansion, affluence, industrialization and urbanization are significant factors influencing economic success, it is inevitable that they will have environmental consequences. These mixed results can be utilized by policymakers to gain insights and provide a framework for future study on potential combinations of factors that may impede or impact the effectiveness of PM2.5 pollution reduction measures and economic policies in SAARC countries.

## Conclusion

Environmental deterioration is caused by a combination of human activity and natural processes. The country’s swift advancement has led to a substantial need for energy resources, concomitant with noteworthy shifts in human behavior. The objective of this study was to examine the primary elements that contribute to the occurrence of haze pollution. This research utilized various econometric methodologies, such as the cross-sectional dependence (CD) test, slope heterogeneity test, unit root test, first and second-generation tests for long-term cointegrating relationships, panel two-stage least squares (TSLS) and Feasible Generalized Least Squares (FGLS) test for robustness and Dumitrescu Hurlin (D-H) panel causality testing. The region is expected to see a rise in PM2.5 pollution due to a combination of factors including rapid economic expansion, heightened consumption expenditures, alterations in forest area, less rainfall and elevated temperatures. Furthermore, the research suggests that the presence of PM2.5 pollution may have the capacity to impact the other factors being examined through a feedback process. The results of the long-term cointegration study indicate, (a) a statistically significant association between haze and the parameters under investigation. (b) The degradation of the environment is mostly driven by the increase of economic activities and the size of forested regions. (c) It is found that an elevation in precipitation levels and average temperature will have a significant mitigating effect on haze pollution. The findings of the causality test suggest that (d) the manipulation of PM2.5 concentrations will have a detrimental impact on a minimum of two, if not all, of the variables. The findings of the study indicate that (e) the concurrent occurrence of expanding economic expansion and diminishing forest coverage would result in a subsequent escalation of PM2.5 concentrations. It is important to thoroughly analyze prospective future scenarios, since they possess the ability to diverge from the present ones under examination.

Similar to other advanced and developed nations, SAARC countries will unavoidably be required to undergo an economic revolution. Modifications in economic conditions have the potential to alter the causal linkages that exist between PM2.5 pollution and several other factors. It is recommended that the mitigation of PM2.5 pollution be accomplished by implementing strategies such as regional precipitation augmentation, stability of temperature fluctuations and directing consumer expenditures towards ecologically sustainable products. This study recognises the expected economic expansion in South Asian countries (SAARC) and the possibility of this changing the connection between PM2.5 pollution and other environmental factors. Nevertheless, addressing air pollution requires more than just slowing economic growth. Instead, it is important to shift our attention towards a sustainable development model that places equal importance on environmental well-being and economic progress. Governments and leaders in SAAC countries must accord utmost priority to the execution of comprehensive policies that are grounded in existing connections, in order to foresee future trends successfully. When formulating pollution mitigation strategies, policymakers should take into account the effects of PM2.5 and other pertinent variables. Stakeholders may endeavor to do the below measures with the aim of formulating an all-encompassing policy. (1) Fostering green energy and consumer choices: giving priority to green energy sources and implementing policies that promote consumer spending on eco-friendly products. This promotes a shift away from fossil fuels and encourages a more sustainable approach to consumption. (2) Environmental awareness and education: creating and executing public awareness campaigns and educational initiatives to empower individuals to make informed decisions that benefit the environment. Having a well-educated public can make a significant impact on reducing pollution. Promote and implement environmental awareness and practices in various economic sectors, empowering individuals to become environmentally conscious citizens. (3) Temperature stabilization: implementing energy frameworks and policies that encourage the expansion of vegetation and forestry as effective measures. Implementing this solution can effectively reduce temperature fluctuations, thereby minimizing the potential impact on PM2.5 levels. Through the implementation of these thorough policies that consider the interplay between economic and environmental factors, SAARC countries can successfully attain sustainable development while addressing the issue of PM2.5 air pollution. To establish the sustainable development framework, stakeholders must separate themselves from generating more PM2.5 pollutant.

It is important to acknowledge, yet, that the research possesses many constraints. The study employs the country as the geographical unit, which constitutes a somewhat expansive scale of spatial analysis. The degree of haze pollution exhibits variability among cities and nations throughout SAARC, leading to significant degrees of variation within the area. A diverse range of tests was employed to account for the heterogeneity of the panel and mitigate the issue of cross-sectional reliance. Nevertheless, there are a number of obstacles that must be addressed in order to get comprehensive and enduring data on PM2.5 concentrations, socio-economic and anthropogenic factor at the city level. Therefore, the findings of this study will provide a foundation for future investigations, contingent upon the accessibility of data such as PM2.5 measurements obtained from a proximate monitoring facility and other relevant factors. Future study should investigate the potential geographical associations between PM2.5 pollution and other pertinent variables, such as the expansion of heavy industry, the density of traffic, the number of exports and the levels of dust pollution.

## Data Availability

The [monthly average temperature, total monthly rainfall] data that support the findings of this study are available at [WorldClim database], [https://www.worldclim.org/data/monthlywth.html]; The [yearly average PM2.5 concentration] data that support the findings of this study are availed from [Atmospheric Composition Analysis Group (ACA)], [https://sites.wustl.edu/acag/datasets/surface-pm2-5/]; The other variable [economic growth] data are available at [World Development Indicators], [https://www.macrotrends.net/]; The other variable [consumption expenditure and forest area] data are available at [World Development Indicators], [http://data.worldbank.org].
